# Methylglyoxal inhibits nuclear division through alterations in vacuolar morphology and accumulation of Atg18 on the vacuolar membrane in *Saccharomyces cerevisiae*

**DOI:** 10.1038/s41598-020-70802-8

**Published:** 2020-08-17

**Authors:** Wataru Nomura, Miho Aoki, Yoshiharu Inoue

**Affiliations:** 1 Laboratory of Molecular Microbiology, Division of Applied Life Sciences, Graduate School of Agriculture, Kyoto University, Uji, Kyoto, 611-0011 Japan; 2 Present Address: Laboratory of Molecular Function of Food, Division of Food Science and Biotechnology, Graduate School of Agriculture, Kyoto University, Uji, Kyoto, 611-0011 Japan

**Keywords:** Cellular microbiology, Stress signalling

## Abstract

Methylglyoxal (MG) is a natural metabolite derived from glycolysis, and it inhibits the growth of cells in all kinds of organisms. We recently reported that MG inhibits nuclear division in *Saccharomyces cerevisiae*. However, the mechanism by which MG blocks nuclear division remains unclear. Here, we show that increase in the levels of phosphatidylinositol 3,5-bisphosphate (PtdIns(3,5)*P*_2_) is crucial for the inhibitory effects of MG on nuclear division, and the deletion of PtdIns(3,5)*P*_2_-effector Atg18 alleviated the MG-mediated inhibitory effects. Previously, we reported that MG altered morphology of the vacuole to a single swelling form, where PtdIns(3,5)*P*_2_ accumulates. The changes in the vacuolar morphology were also needed by MG to exert its inhibitory effects on nuclear division. The known checkpoint machinery, including the spindle assembly checkpoint and morphological checkpoint, are not involved in the blockade of nuclear division by MG. Our results suggest that both the accumulation of Atg18 on the vacuolar membrane and alterations in vacuolar morphology are necessary for the MG-induced inhibition of nuclear division.

## Introduction

The inheritance of chromosomes is a critical biological event for all organisms. Eukaryotic cells contain chromosomal DNA in the nucleus, an organelle enveloped in a double lipid bilayer (nuclear membrane). Unlike higher eukaryotes, the nuclear membrane in yeast cells is not degraded during mitosis (closed mitosis); therefore, the nucleus is transported to the daughter cell during the mitotic process^1^. The budding yeast *Saccharomyces cerevisiae* is excellent for observing this process. A bud (daughter cell) begins to emerge from the mother cell at S phase and grows larger at M phase. The nuclear membrane is extended, a portion of the nucleus penetrates the bud, and then the nucleus is separated in the late M phase. Consequently, a set of chromosomes is inherited to the bud^2^.

Methylglyoxal (MG) is a typical 2-oxoaldehyde derived from glycolysis^3–5^. Despite being a natural metabolite, MG at high concentrations inhibits the growth of cells in all types of organisms; however, precisely how it exerts its toxicity is unclear. Since MG enhances the frequency of sister chromatid exchange and endoreduplication in CHO-AUXBI cells^6^, it may have some effect on chromatin maintenance and inheritance. However, treatment with MG causes cell cycle arrest at G1 phase in human HL60 cells. Meanwhile, Kani et al.^7^ have reported that MG causes oxidative stress, thereby inducing G2/M arrest in HEK293 cells. However, whether treatment with MG can elicit oxidative stress depends on the cell lines used; therefore, it is unclear if MG per se is the cause of cell cycle arrest. We have reported that MG does not cause oxidative stress in *S*. *cerevisiae*, although at moderate concentrations it causes temporal growth arrest without decreasing the cell viability^8^. In addition, we recently demonstrated that the depolarization of the actin cytoskeleton and blockade of the nuclear division by MG treatment are the reasons for MG toxicity^9,10^. These phenotypes were also observed when a mutant defective in MG metabolizing enzymes was treated with dihydroxyacetone, which caused an increase in the intracellular levels of MG^10^. Therefore, intracellular MG seems to be involved in the inhibition of nuclear division; however, the mechanism by which MG blocks the transportation of nucleus to the daughter cells remains unknown.

Our recent study on the screening of MG-sensitive mutants showed that the mutants defective for the synthesis pathway of phosphatidylinositol 3,5-bisphosphate (PtdIns(3,5)*P*_2_), a minor species of phosphoinositide, exhibited higher susceptibility to MG^11^. MG increases the levels of PtdIns(3,5)*P*_2_ and simultaneously induces morphological changes in the vacuoles in which PtdIns(3,5)*P*_2_ is predominantly distributed^11^. In this study, we show that the alterations in the vacuolar morphology and increase in the levels of PtdIns(3,5)*P*_2_ are required for the MG-induced inhibition of nuclear division, and the PtdIns(3,5)*P*_2_ effector protein Atg18 is involved in the inhibitory machinery. The checkpoint machineries involved in the cell cycle arrest at G2/M in *S. cerevisiae*, i.e. the spindle assembly checkpoint and morphological checkpoint, were not crucial for the MG-induced inhibition of nuclear division. These results indicate that alterations in the vacuolar morphology and accumulation of Atg18 on the vacuolar membrane are necessary events for the MG-induced inhibition of nuclear division, which occurs independently of the activation of known checkpoints.

## Results

### Effector of PtdIns(3,5)*P*_2_, Atg18, is necessary for the MG-induced inhibition of nuclear division

**Figure 1:**
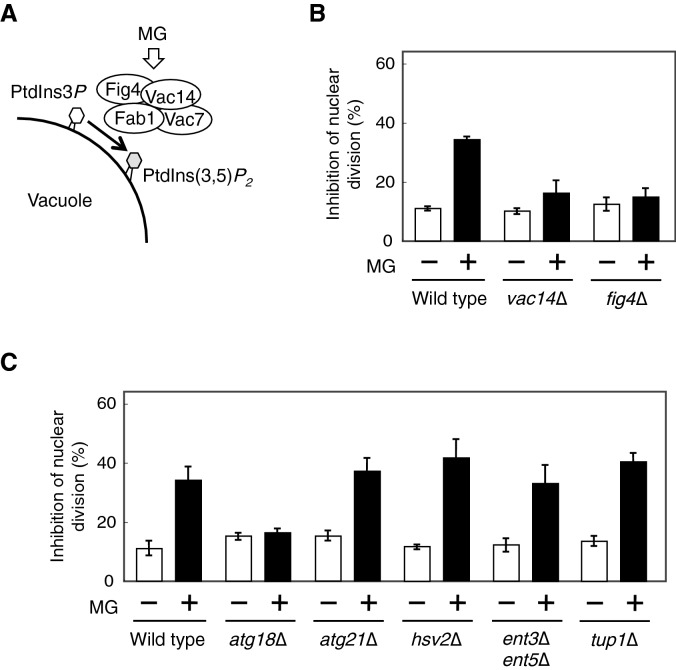
Atg18 is involved in MG-induced inhibition of nuclear division. (**A**) Model of PtdIns(3,5)*P*_*2*_ synthesis on vacuolar membrane by Fab1 complex. MG activates the pathway of PtdIns(3,5)*P*_*2*_ synthesis. (**B**) Wild type, *vac14*∆, and *fig4*∆ cells were cultured in SD medium till *A*_610_ = 0.3–0.5, and were treated with 10 mM MG for 90 min. The inhibition rate of nuclear division was determined by evaluating the status of the nuclei (stained with Hoechst 33342) in cells having a large bud (bud diameter approximately two-thirds of that of the mother cell). Data are from three independent experiments (mean ± standard deviation), and more than 100 cells were counted for each experiment. (**C**) Wild type, *atg18*∆, *atg21*∆, *hsv2*∆, *ent3*∆*ent5*∆, and *tup1*∆ cells were cultured in SD medium until *A*_610_ = 0.3–0.5, and were treated with 10 mM MG for 90 min. The inhibition rate of nuclear division was determined as described in (**B**).

MG blocks the nuclear division and induces an accumulation of undivided nuclei in the wild type cells^10^ (Fig. 1B). To identify the mechanism involved in the inhibitory effects of MG on nuclear division, we focused on the involvement of PtdIns(3,5)*P*_2_ because its levels are increased by MG treatment. Although the total cellular abundance of PtdIns(3,5)*P*_2_ is low, it is predominantly distributed in the vacuolar membrane^12^, and is involved in the maintenance of vacuolar morphology and its functions^12–17^. The synthesis of PtdIns(3,5)*P*_2_ is catalysed by PtdIns3*P* 5-kinase encoded by *FAB1*, and Fab1 interacts with some its regulators, Vac7, Vac14, and Fig4, which together form the Fab1 complex^14,18–20^ (Fig. 1A). MG increases the levels of PtdIns(3,5)*P*_2_ at the vacuolar membrane in a Fab1 complex-dependent manner^11^. We examined whether the increase in the levels of PtdIns(3,5)*P*_2_ is involved in the MG-induced inhibition of nuclear division. The results obtained showed that the nuclear division was hardly inhibited in the Fab1 complex mutants*, vac14*∆ and *Fig4*∆, in the presence of MG (Fig. 1B), suggesting that the increase in the levels of PtdIns(3,5)*P*_2_ is required for the MG-induced inhibition of nuclear division.

To date, 6 PtdIns(3,5)*P*_2_-binding proteins (Atg18, Atg21, Hsv2, Ent3, Ent5, and Tup1) have been identified in *S. cerevisiae*^12,21–26^. Ent3 and Ent5 have redundant functions. Since an increase in the levels of PtdIns(3,5)*P*_2_ following treatment with MG appeared to be involved in the inhibition of nuclear division (Fig. 1B), we determined whether these PtdIns(3,5)*P*_2_ effectors participated in the MG-induced accumulation of undivided nuclei. As shown in Fig. 1C, the deletion of *ATG18* suppressed the inhibitory effects of MG on nuclear division, whereas those of *ATG21*, *HSV2*, *ENT3*, *ENT5*, and *TUP1* did not.

### Effect of mutated Atg18 localizing artificially at the vacuole on the blockade of nuclear division

**Figure 2:**
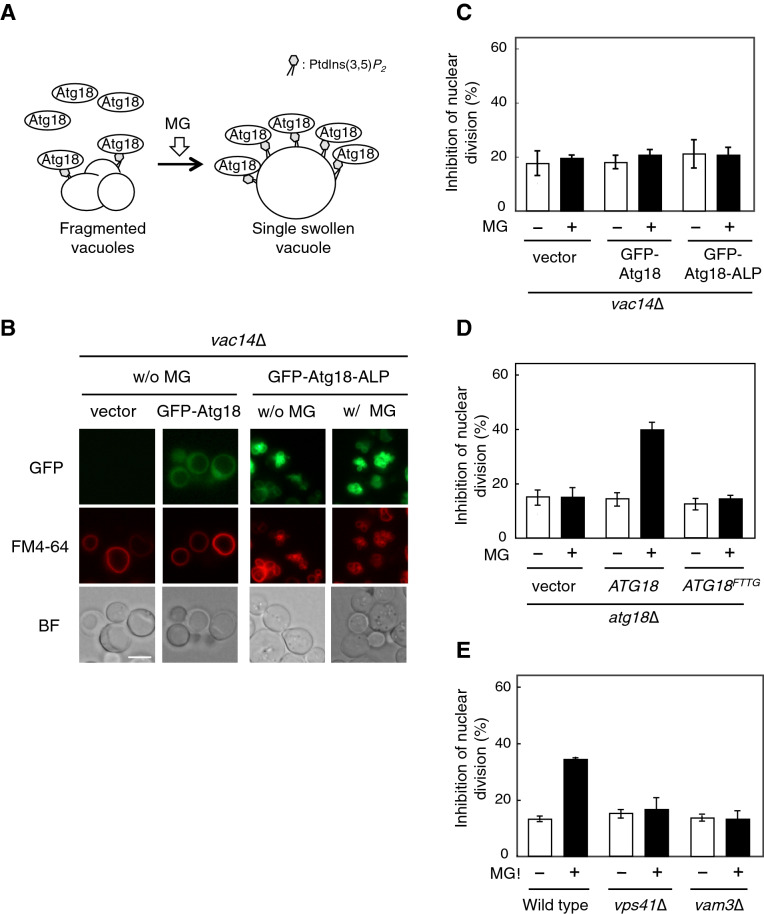
Effect of mutated Atg18 localizing artificially at vacuole on the blockade of nuclear division. (**A**) Model showing that MG facilitates the accumulation of Atg18 on the vacuolar membrane and causes alterations in the vacuolar morphology. (**B**) *vac14*∆ cells carrying pRS415 (vector), pRS415-MET25p-GFP-Atg18 (GFP-Atg18), or pRS415-MET25p-GFP-Atg18-ALP (GFP-Atg18-ALP) were cultured in SD medium until *A*_610_ = 0.3–0.5. Atg18-GFP and the morphology of the vacuole (FM4-64) were observed using a fluorescence microscope. Bar, 5 µm. (**C**) *vac14*∆ cells carrying an empty vector, GFP-Atg18, or GFP-Atg18-ALP were cultured in SD medium until *A*_610_ = 0.3–0.5, and were treated with 10 mM MG for 90 min. The rate of nuclear division was determined as described in the legend for Fig. 1B. (**D**) *atg18*∆ cells carrying an pRS416 (vector), pRS416-*ATG18* or pRS416-*ATG18*^*FTTG*^ were cultured in SD medium until *A*_610_ = 0.3–0.5, and were treated with 10 mM MG for 90 min. The rate of nuclear division was determined as described in the legend for Fig. 1B. (**E**) Wild type, *vps41*∆, and *vam3*∆ cells were cultured in SD medium until *A*_610_ = 0.3–0.5, and were treated with 10 mM MG for 90 min. The inhibition rate of nuclear division was determined as described in Fig. 1B.

Atg18 is well known as a core component for the vesicle formation during autophagy^27^ and its role in the regulation of vacuole fission^28^. In the microscopy studies with GFP fusion protein, Atg18 was observed in the limited area of the vacuolar membrane as well as in the punctate structures^22,29,30^. We have shown that MG enhanced the accumulation of Atg18 on the vacuolar membrane in accordance with the increase in the levels of PtdIns(3,5)*P*_2_, and this effect was abrogated in *vac14*∆ cells^10,11^ (Fig. 2A). These findings raised the possibility that the recruitment of Atg18 to the vacuolar membrane by an increasing concentration of PtdIns(3,5)*P*_2_ in the vacuolar membrane is the cause of MG-induced inhibition of nuclear division. Hence, the suppressive effects observed in the mutants of PtdIns(3,5)*P*_2_, with decreased synthesis of PtdIns(3,5)*P*_2,_ on the inhibition of nuclear division might be abrogated when Atg18 can be artificially targeted to the vacuolar membrane. It is reported that a fusion protein GFP-Atg18-ALP is constitutively associated with the vacuole^29^. We then examined whether the inhibition of nuclear division is induced by expressing GFP-Atg18-ALP in *vac14*∆ cells, where PtdIns(3,5)*P*_2_ is hardly detected. As shown in Fig. 2B, GFP-Atg18-ALP localized at the vacuolar membrane in *vac14*∆ cells, although the accumulation of GFP-Atg18 on the vacuolar membrane was not clearly observed. The vacuolar localization of GFP-Atg18-ALP was not changed following the treatment with MG (Fig. 2B). In *vac14*∆ cells expressing GFP-Atg18-ALP, the proportion of cells with undivided nuclei did not significantly increase in the presence of MG (Fig. 2C), indicating that the suppressive effects observed in the mutants of PtdIns(3,5)*P*_2_ synthesis on the inhibition of nuclear division were not abrogated by the artificial localization of Atg18 to the vacuolar membrane. Atg18 binds to phosphoinositides via the conserved FRRG motif, and mutation of the FRRG motif in Atg18, Atg18^FTTG^, abolishes the capability of binding to PtdIns(3,5)*P*_2_, which loses its localization to the vacuolar membrane and punctate structures^31^. We further verified whether the binding of Atg18 to PtdIns(3,5)*P*_2_ is necessary for its contribution to the MG-induced inhibition of nuclear division using an Atg18^FTTG^ mutant. As shown in Fig. 2D, Atg18^FTTG^ was not able to revert the suppressive effect of MG on the nuclear division in *atg18*∆ cells. These results suggest that the accumulation of Atg18 on the vacuolar membrane through the binding to PtdIns(3,5)*P*_2_ is necessary for the MG-induced inhibition of nuclear division.

### Changes in vacuolar morphology are involved in the MG-induced inhibition of nuclear division

Wild type cells predominantly have 2–4 fragmented vacuoles, while *vac14*∆ mutant cells have a single grossly enlarged vacuole^18,19^. In Fig. 2B, the expression of GFP-Atg18-ALP led to a striking fragmentation of the vacuole, and this phenotype was consistent with that shown in a previous study^29^. We recently showed that MG changed the vacuolar morphology to a single swelling form^11^ (Fig. 2A), and the MG-induced vacuolar swelling did not occur in *vps41*∆ and *vam3*∆ mutants, in which the vacuolar fusion is defective^11,32^. These findings raise the possibility that the changes in the vacuolar morphology are also necessary for the MG-induced inhibition of nuclear division. Therefore, we investigated the effect of MG on nuclear division in these mutants. As shown in Fig. 2E, the proportion of cells which possess undivided nuclei did not significantly increase after treatment with MG in *vps41*∆ and *vam3*∆ cells, suggesting that the vacuolar swelling in addition to an increase in PtdIns(3,5)*P*_2_ and the vacuolar localization of Atg18 is a prerequisite for the MG-induced inhibition of nuclear division.

### MG does not affect the formation of microtubules or duplication of spindle pole bodies

**Figure 3:**
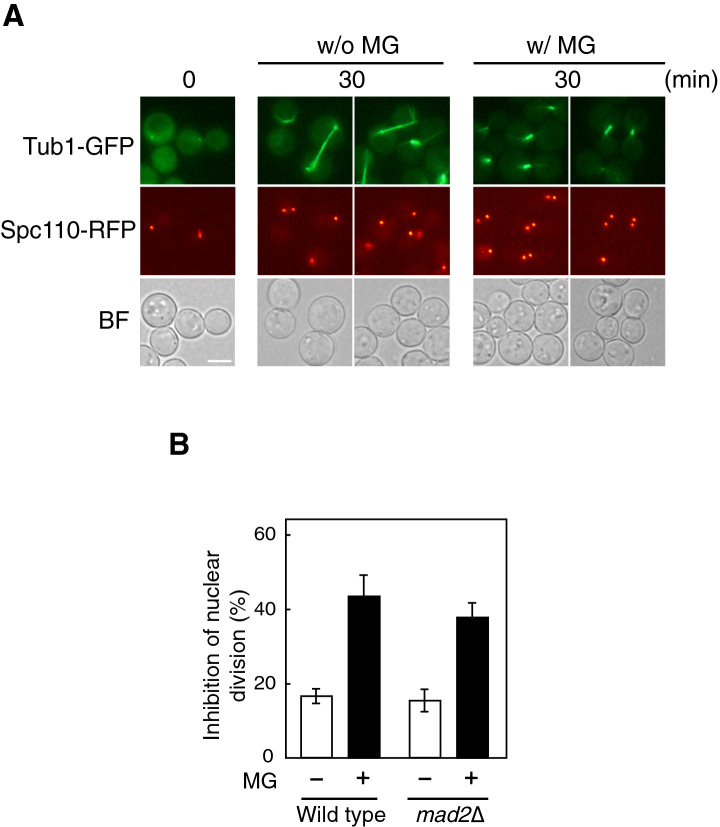
Effect of MG on microtubule organization. (**A**) Cells (YPH250) carrying both *TUB1-GFP* and *SPC110-RFP* were cultured in SD medium until *A*_610_ = 0.3, harvested by centrifugation, and suspended in YPD medium containing 6 µg/ml nocodazole. After 180 min, cells were suspended in fresh SD medium, with or without 10 mM MG. After 30 min, SPB (Spc110-RFP) and microtubules (Tub1-GFP) were observed using a fluorescence microscope. Bar, 5 µm. (**B**) The wild-type (YPH250) and *mad2*∆ mutant cells were cultured in SD medium until *A*_610_ = 0.3–0.5, and were treated with 10 mM MG for 90 min. The inhibition rate of nuclear division was determined as described in the legend of Fig. 1B.

We recently reported that in association with the inhibition of nuclear division, the nuclear morphology changed from a globate shape to one with a central depression aligned with the mother-bud axis, which we refer as a “jellybean-like shape” of the nucleus, following treatment with MG^10^. The jellybean-like shaped nucleus did not enter into the bud growing larger because MG seemed to have arrested the cell cycle at the G2/M transition with respect to the nuclear division^10^. Microtubules play a key role in the nuclear dynamics throughout the cell cycle^33,34^. In *S*. *cerevisiae*, spindle pole bodies (SPBs) are the microtubule-organizing centres that are necessary for the nucleation and organization of microtubule arrays^35^. Since *S. cerevisiae* undergoes closed mitosis, SPBs are embedded in the nuclear envelope throughout the cell cycle. SPBs are duplicated by a conservative mechanism at G1/S transition. Cells in preanaphase and anaphase contain two SPBs aligned with the mother-bud axis, which defines the direction of nuclear division. The nuclear microtubules (spindles) are organized toward the nucleus from SPBs. So, we determined the effects of MG on the formation of SPBs and microtubules. Cells in the early log phase were treated with nocodazole for 180 min to collapse the microtubules, and were then released to fresh medium with or without MG. We observed SPBs and microtubules using RFP (DsRed)-tagged Spc110, an inner plaque SPB component, and GFP-tagged Tub1, α-tubulin, respectively. As shown in Fig. 3A, SPBs were duplicated after 30 min of releasing cells to fresh medium without MG, and telophase spindle elongation was observed as the consequence of nuclear division. The duplication of SPBs and spindle formation and its orientation (aligned with the mother-bud axis) were normal in the presence of MG (Fig. 3A); however, the length of spindles was shorter because of the inhibition of nuclear division. These results suggest that MG is unlikely to affect the formation of spindles or duplication of SPBs.

The spindle assembly checkpoint ensures proper attachment between the spindles and kinetochores, and controls the fidelity of chromosome segregation^36^. Hence, entry into anaphase is inhibited when spindles do not attach properly to the kinetochores. Mad2 is a component of the spindle assembly checkpoint complex that regulates entry of the cell cycle into anaphase^36^; therefore, cell cycle proceeds in *mad2*∆ cells even though spindles are not constructed adequately. As shown in Fig. 3B, the inhibition of nuclear division occurred following the treatment with MG, even in *mad2*∆ cells, suggesting that MG neither inhibits spindle formation nor activates the spindle assembly checkpoint.

### The expression of constitutively active allele of *PKC1* or the disruption of *SWE1* alleviates the MG-induced inhibition of nuclear division

**Figure 4:**
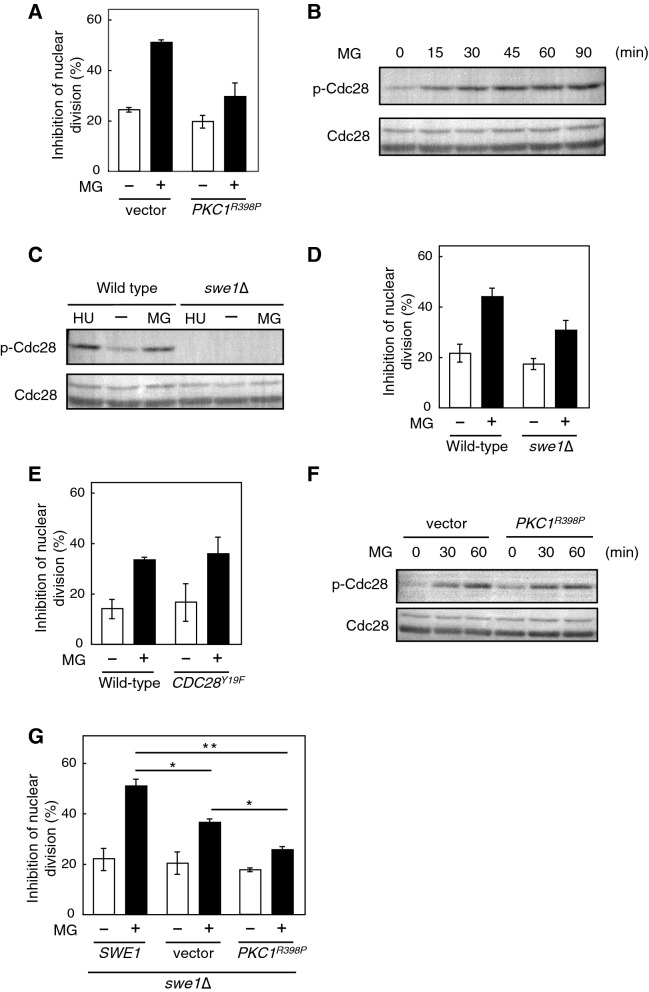
Effect of MG on phosphorylation of Cdc28. (**A**) Cells (YPH250) carrying YCp50 (vector) or YCp50-*PKC1*^*R398P*^ were cultured in SD medium until *A*_610_ = 0.3, and were treated with 10 mM MG for 90 min. The inhibition rate of nuclear division was determined as described in Fig. 1B. (**B**) Cells (DLY1) were cultured in SD medium until *A*_610_ = 0.3 and were treated with 10 mM MG for the prescribed time indicated in the figure. Phosphorylation levels of Cdc28 (p-Cdc28) and total protein expression levels of Cdc28 were determined using anti-phospho (Tyr19) Cdc28 antibodies and anti-Cdc2 antibodies, respectively. (**C**) Wild type (DLY1) and *swe1*∆ mutant (DLY1028) cells were cultured in SD medium until *A*_610_ = 0.3, and were treated with 10 mM MG for 30 min or 100 mM hydroxyurea (HU) for 120 min. The phosphorylation of Cdc28 was determined as described in (**B**). (**D**) Wild type (DLY1) and *swe1*∆ mutant (DLY1028) cells were cultured in SD medium until *A*_610_ = 0.3, and were treated with 10 mM MG for 90 min. The inhibition rate of nuclear division was determined as described in Fig. 1B. (**E**) Wild type (W303-1B) and *CDC28*^*Y19F*^ mutant cells were cultured in SD medium until *A*_610_ = 0.3, and were treated with 10 mM MG for 90 min. The inhibition rate of nuclear division was determined as described in Fig. 1B. (**F**) Cells (DLY1) carrying YCp50 (vector) or YCp50-*PKC1*^*R398P*^ at an early log-phase of growth were treated with 10 mM MG for the prescribed time as indicated in the figure, and the phosphorylation of Cdc28 was determined as described in (**B**). (**G**) *swe1*∆ cells (DLY1028) carrying YCp50 (vector), pKL2698, or YCp50-*PKC1*^*R398P*^ were treated with MG as described in (**A**). The inhibition rate of nuclear division was determined as described in Fig. 1B. *p < 0.05; **p < 0.01.

*S. cerevisiae* undergoes polarized growth, and the establishment of cell polarity that indicates the direction from the mother to the bud is crucial for it^37^. Actin cytoskeleton is important for the establishment of cell polarity, and thereby a transportation of organelles to daughter cell during the polarized growth is warranted^37^. A morphological checkpoint monitors actin organization^38,39^, and filamentous actin disorganization leading to the morphological checkpoint causes the phosphorylation of Tyr19 in Cdc28, a budding yeast homologue of the cyclin-dependent kinase Cdc2 that controls the timing of entry into mitosis^38–41^. Phosphorylation of Cdc28 at Tyr19 lowers the activity of the G2 cyclin-Cdc28 complex, which leads to the inhibition of entry into mitosis; consequently, the nuclear division is interrupted^2^. Therefore, the activation of the morphological checkpoint induces G2/M cell cycle arrest^38–40^. We previously reported that MG induced the depolarization of actin patches, and the expression of a constitutively active allele of *PKC1* (*PKC1*^*R398P*^) was partially able to repress the depolarization of the actin patches in cells treated with MG^9^. As shown in Fig. 4A, an increase in the proportion of cells whose nuclei were neither divided nor transported into the bud following treatment with MG was suppressed when *PKC1*^*R398P*^ was introduced. We then explored the possibility that the morphological checkpoint participates in the MG-induced inhibition of nuclear division. To investigate this possibility, we determined the phosphorylation of Cdc28 at Tyr19 in cells treated with MG. As shown in Fig. 4B, phosphorylation occurred after 15 min of MG treatment. Hydroxyurea (HU) is well known to enhance the phosphorylation of Cdc28^42^. The phosphorylation of Cdc28 is catalysed by the protein kinase Swe1, a homologue of *S. cerevisiae* Wee1^43^. As expected, the phosphorylation of Cdc28 did not occur following treatment with MG in *swe1*∆ cells (Fig. 4C), indicating that MG causes the phosphorylation of Cdc28 at Tyr19 in a Swe1-dependent manner.

Next, we determined whether the disruption of *SWE1* alleviates the inhibitory effect of MG on nuclear division. As shown in Fig. 4D, the inhibitory effect of MG on the nuclear division in *swe1*∆ was slightly lower than that in wild type cells; however, the proportion of cells in which nuclear division was inhibited in the presence of MG did not decrease in cells carrying *CDC28*^*Y19F*^, a non phosphorylatable allele of *CDC28* (Fig. 4E). The phosphorylation of Cdc28 occurred following the treatment with MG, even in cells introduced with *PKC1*^*R398P*^ (Fig. 4F). Furthermore, an additive effect between the expression of *PKC1*^*R398P*^ and the disruption of *SWE1* for the alleviation of MG-induced inhibition of nuclear division was observed (Fig. 4G). When the wild type allele of *SWE1* was reverted in the *swe1*∆ mutant, the proportion of cells with inhibited nuclear division was increased (*swe1*∆/*SWE1*: w/o MG, 22 ± 4%; w/ MG, 51 ± 3%) compared with that in *swe1*∆ cells carrying the vector alone (*swe1*∆/vector: w/o MG, 20 ± 4%; w/ MG, 37 ± 1%), and when *PKC1*^*R398P*^ was expressed in *swe1*∆ cells, the restoration of nuclear division that had been inhibited by MG occurred at a much greater extent compared with that in *swe1*∆ cells carrying the vector; i.e. the proportion of cells with inhibited nuclear division did not increase as much (*swe1*∆/*PKC1*^*R398P*^: w/o MG, 18 ± 1%; w/ MG, 26 ± 1%) compared with the vector control. These results indicate that the phosphorylation of Cdc28 at Tyr19 does not commit to the MG-induced inhibition of nuclear division; the expression of *PKC1*^*R398P*^ and the disruption of *SWE1* independently contribute to the alleviation of inhibitory effect of MG on the nuclear division.

### The expression of *PKC1*^*R398P*^ alleviates the MG-induced vacuolar swelling

**Figure 5:**
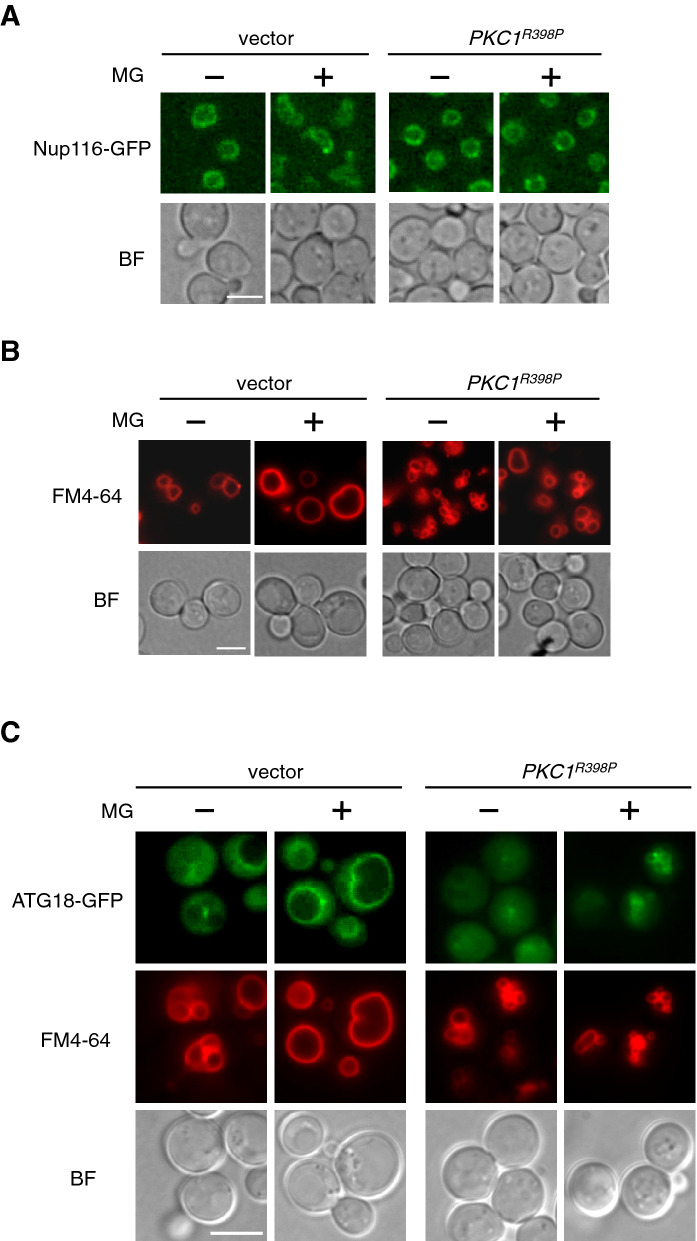
Effect of the expression of *PKC1*^*R398P*^ on vacuolar morphology. (**A**) Cells (YPH250) carrying *NUP116-GFP* and either YCp50 (vector) or YCp50-*PKC1*^*R398P*^ were cultured in SD medium until *A*_610_ = 0.3 and were treated with 10 mM MG for 90 min. Nup116-GFP was observed using a fluorescence microscope. Bar, 5 µm. (**B**) Cells (YPH250) carrying YCp50 (vector) or YCp50-*PKC1*^*R398P*^ were cultured in SD medium until *A*_610_ = 0.3, and were treated with 10 mM MG for 90 min. The vacuolar membrane was stained with FM4-64. Bar, 5 µm. (**C**) Cells (YPH250) carrying *ATG18-GFP* and either pFL39 (vector) or pFL39-*PKC1*^*R398P*^ were cultured in SD medium until *A*_610_ = 0.3 and were treated with 10 mM MG for 90 min. Atg18-GFP and FM4-64 were observed using a fluorescence microscope. Bar, 5 µm.

MG-induced changes in the nuclear morphology were observed using nuclear membrane-located Nup116-GFP, a component of the nuclear pore complex, to the jellybean-like shape^10^ (Fig. 5A). In *PKC1*^*R398P*^-expressing cells, we noticed that the emergence of the jellybean-like shaped nucleus following treatment with MG was partially repressed (Fig. 5A). The occurrence of the jellybean-like shaped nucleus was caused by MG-induced vacuolar swelling^10^. We then observed the effects of the *PKC1*^*R398P*^ expression on the vacuolar morphology. The *PKC1*^*R398P*^-expressing cells had fragmented vacuoles, and MG-induced vacuolar swelling in this strain was not so obvious as that in the wild type cells (Fig. 5B). The steady state levels of PtdIns(3,5)*P*_2_ were not affected by the expression of *PKC1*^*R398P*^; however, the levels of which were slightly increased in the *PKC1*^*R398P*^-expressed cells following the treatment with MG (Table 1). Consistent with this result, Atg18-GFP accumulated on the fragmented vacuolar membrane following treatment with MG in the *PKC1*^*R398P*^-expressing cells (Fig. 5C). These findings indicate that the vacuolar fragmentation caused by the *PKC1*^*R398P*^ expression is involved in the suppression of the MG-induced inhibition of nuclear division for which the vacuolar swelling is a prerequisite.

**Table 1:** Levels of phosphatidylinositols in *PKC*^*R398P*^ cells.

	**w/o MG**		**w/ MG**	
	**vector**	***PKC1***^***R398P***^	**vector**	***PKC1***^***R398P***^
PtdIns3*P*	0.97 ± 0.03	0.78 ± 0.05	0.96 ± 0.05	0.79 ± 0.03
PtdIns4*P*	1.61 ± 0.05	1.57 ± 0.10	1.53 ± 0.04	1.39 ± 0.09
PtdIns(3,5)*P*_2_	0.023 ± 0.002	0.029 ± 0.014	0.055 ± 0.003	0.076 ± 0.02
PtdIns(4,5)*P*_2_	0.54 ± 0.07	0.54 ± 0.04	0.49 ± 0.08	0.48 ± 0.05

Wild type cells carrying YCp50 (vector) or YCp50-*PKC1*^*R398P*^ were treated with 10 mM MG for 60 min. The extraction and measurement of each phosphatidylinositol are described in the Methods section. The level of each phosphatidylinositol is shown as a percentage of the total levels of phosphatidylinositol (mean ± standard deviation, n = 3).

## Discussion

**Figure 6:**
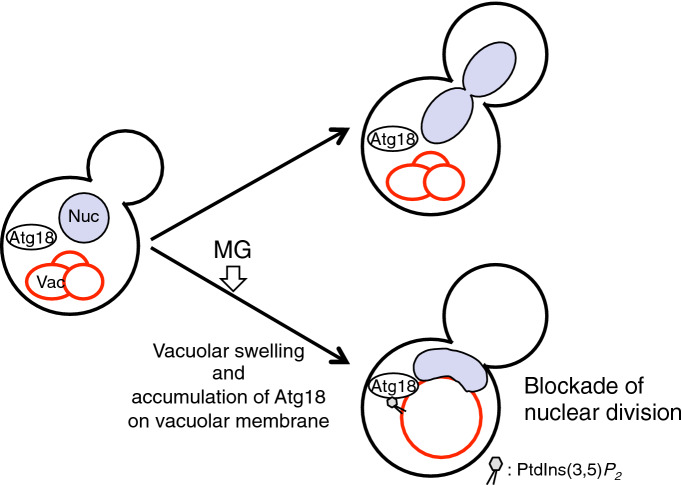
A model for the blockade of nuclear division by MG. Both the increase in levels of PtdIns(3,5)*P*_2_ and vacuolar swelling act as the primary signal for the MG-induced inhibition of nuclear division, and then Atg18 localized to the swollen vacuole through PtdIns(3,5)*P*_2_ commits to the blockage of nuclear division. Under that condition, the nuclear morphology changes to a jellybean-like shape. Nuc, nucleus. Vac, vacuole.

MG inhibits the growth of cells in all organisms that have been examined so far; however, the underlying mechanism has not been fully elucidated. We recently reported that MG blocked the nuclear division in *S. cerevisiae*, implying that the blockade of nuclear division is one of the mechanisms by which MG arrests cell growth^10^. Even though the precise mechanism underlying the MG-induced inhibition of nuclear division has not been uncovered, we have identified that Atg18 is crucial for the inhibitory mechanism (Fig. 1C). In the present study, we have revealed that the inhibitory effect of MG on nuclear division was exerted through vacuolar swelling (Fig. 2E) and increase in the levels of PtdIns(3,5)*P*_*2*_ (Fig. 1B), which facilitated the vacuolar localization of Atg18 (Fig. 6).

Atg18 has a WD-40 repeat motif that shows binding affinity for PtdIns(3,5)*P*_2_^12^. Previously, we reported that MG induces the accumulation of Atg18 at the vacuolar membrane through increased PtdIns(3,5)*P*_2_ levels on the vacuolar membrane^10,11^. In Fig. 2D, the blockade of nuclear division by MG was not observed in the *atg18*∆ cells expressing Atg18^FTTG^ that is unable to bind PtdIns(3,5)*P*_2_ thereby losing its localization to the vacuolar membrane. It seems likely that the accumulation of Atg18 is sufficient for the inhibition of nuclear division; however, the expression of mutated Atg18 that artificially targeted at the vacuolar membrane in a PtdIns(3,5)*P*_2_-independent manner did not enhance the inhibitory effect of nuclear division in *vac14*∆ cells (Fig. 2C), implying that the binding of Atg18 to PtdIns(3,5)*P*_2_ is a key to exert the inhibition of nuclear division. Meanwhile, the expression of mutated Atg18 caused morphological alterations in the vacuoles of *vac14*∆ cells, from a single large form to a fragmented form, which did not change to the swelling form by MG (Fig. 2B)^29^. Therefore, non-significant effect of the expression of mutated Atg18 (Atg18-ALP) on the blockade of nuclear division may be due to the fragmentation of vacuoles, supporting our conclusion that both the accumulation of Atg18 at the vacuolar membrane and vacuolar swelling are necessary for the blockade of nuclear division by MG (Fig. 6). A recent study showed that Atg18 contributes to vacuolar fission^28^, which may be the reason for the striking vacuolar fragmentation caused by the expression of vacuolar-localized mutant of Atg18. Atg18 is well known as a core component contributing to the vesicle formation during autophagy processes^27^; however, MG did not induce macroautophagy and micronucleophagy^44^. Further investigations are needed to determine the molecular mechanism of Atg18-mediated inhibitory effects on nuclear division.

Upon MG-induced stress, cell cycle seems to be arrested at the G2/M phase in terms of nuclear division. Indeed, Tyr19 of Cdc28 was phosphorylated following the treatment with MG (Fig. 4B). Two checkpoints have so far been identified that can cause cell cycle arrest at the G2/M phase in *S. cerevisiae*: a spindle assembly checkpoint and a morphological checkpoint^36,38,39^. Nocodazole activates the spindle assembly checkpoint through depolymerization of microtubules, thereby arresting the cell cycle at G2/M. In our study using the *mad2*∆ mutant, the spindle assembly checkpoint was not likely to be activated following treatment with MG (Fig. 3B). Meanwhile, when nocodazole-treated cells were released into fresh medium, the spindle was reorganized rapidly and immediately the cell cycle entered anaphase because DNA synthesis was completed during the period of nocodazole treatment. Consequently, cell cycle proceeded to telophase within 30 min after the release into the fresh medium (Fig. 3A). However, when nocodazole-treated cells were released into the MG-containing medium, cells in preanaphase, i. e. cells with G2-like short spindle and bud-bound SPB being not able to enter the bud, were accumulated even though the spindle exists at the bud neck, and the bud is large enough to accept the nucleus. Therefore, MG may influence some steps for the bud-bound SPB to cross the boundary of the bud neck.

Latrunculin A (Lat-A) activates the morphological checkpoint^38,39^. When *S. cerevisiae* cells were treated with Lat-A, F actin was depolymerized within 15 min, which in turn served as a signal to phosphorylate Tyr19 of Cdc28 to arrest the cell cycle at G2/M^40^. However, phosphorylation of Cdc28 occurred 120 min after treatment with Lat-A. Meanwhile, MG depolarized the actin patches from the buds in 30 min^9^; however, phosphorylation of Cdc28 at Tyr19 preceded (~ 15 min) the depolarization of the actin patches (Fig. 4B). The Lat-A-induced G2 arrest is dependent upon Mpk1 mitogen-activated protein (MAP) kinase cascade^40^. We previously reported that MG induced the activation of Mpk1 MAP kinase cascade^9^, and the activation of Mpk1 MAP kinase cascade did not occur by MG derived from dihydroxyacetone, which increases the intracellular MG^10^, suggesting that the Mpk1 MAP kinase cascade is activated by extracellular MG. However, MG derived from dihydroxyacetone blocks nuclear division^10^, implying that the activation of Mpk1 MAP kinase cascade does not commit to the MG-induced inhibition of nuclear division. Since the mode of action of MG and Lat-A is different, i. e. MG depolarizes the actin cytoskeleton but does not depolymerize F actin whereas Lat-A depolymerizes the actin polymer, the morphological checkpoint may not be activated upon MG stress. In addition, the MG-induced inhibition of nuclear division occurred in *CDC28*^*Y19F*^ mutant cells in which the morphological checkpoint was bypassed (Fig. 4E). Therefore, MG-induced phosphorylation at Tyr19 of Cdc28 is not involved in the inhibition of nuclear division.

As an explanation for the attenuation of the blockade of nuclear division by MG in the *PKC1*^*R398P*^-expressing cells, we found that MG could not cause vacuolar swelling in these cells (Fig. 5B). We reported earlier that the vacuolar swelling is necessary for the MG-induced morphological change in the nucleus to a jellybean-like shape^10,11^. We found that the emergence of the jellybean-like shape of the nucleus following treatment with MG was partially repressed in *PKC1*^*R398P*^-expressing cells (Fig. 5A), implying that the change in nuclear morphology to a jellybean-like shape contributes toward the blockade of nuclear division. Regarding the MG-induced inhibition of nuclear division, we would infer that it may not be a surveillance system but may imply the existence of “nuclear morphology checkpoint”. Further analysis of the MG-induced inhibition of nuclear division might lead to identification of the novel checkpoint mechanism.

## Methods

### Media and reagents

The media used was SD (2% glucose, 0.67% yeast nitrogen base without amino acids) with appropriate amino acids and bases being added wherever necessary. MG was purchased from Sigma-Aldrich (MO, USA). When the *A*_610_ of the culture reached 0.3–0.5, 10 mM MG was added and the cells were incubated at 28ºC with reciprocal shaking. We verified that the cell growth was temporally arrested in the presence of 10 mM MG, while cell viability was maintained.

### Strains

The yeast strains used in the present study are listed in Table 2. The *S. cerevisiae* strains used had the YPH250, DLY1, and W303-1B backgrounds. Deletion mutants of *ATG18*, *ATG21*, *HSV2*, *ENT3*, *ENT5*, *TUP1*, and *MAD2* were constructed by PCR-based methods with *KanMX* or *his5*^+^ selection markers^45^. Deletion constructs were amplified by PCR from BY4741-based deletion mutants (Invitrogen, Carlsbad, CA, USA).

**Table 2:** List of *Saccharomyces cerevisiae* strains used in the present study.

Strain	Relevant genotype/description	Source/References
YPH250 *vac14*∆ *Figure **4*∆ *atg18*∆ *atg21*∆ *hsv2*∆ *ent3*∆*ent5*∆ *tup1*∆ *mad2*∆ *vps41*∆ *vam3*∆ Atg18-GFP DLY1 DLY1028 W303-1B *CDC28*^*Y19F*^	*MATa* *trp1*-∆*1 his3*-∆*200 leu2*-∆*1 lys2-801 ade2-101 ura3-52* YPH250, *vac14*∆::*his5*^+^ YPH250, *fig4*∆::*LEU2* YPH250, *atg18*∆::*his5*^+^ YPH250, *atg21*∆::*KanMX4* YPH250, *hsv2*∆::*KanMX4* YPH250, *ent3*∆::*his5*^+^ *ent5*∆::*KanMX4* YPH250, *tup1*∆::*KanMX4* YPH250, *mad2*∆::*KanMX4* YPH250, *vps41*∆::*KanMX4* YPH250, *vam3*∆::*KanMX4* YPH250, *ATG18-GFP*::*URA3* *MATa* *ade1 his2 leu2-3, 112 trp1-1*^*a*^* ura3ns bar1* DLY1, *swe1*∆::*LEU2* *MAT*α *leu2*-*3*, *112 trp1*-*1 can1-100 ura3-1 ade2-1 his3*-*11*, *15* W303-1B, *CDC28*^*Y19F*^::*HIS3*	Lab stock ^11^ ^11^ This study This study This study This study This study This study ^11^ ^11^ ^11^ ^39^ ^39^ ^53^ NBRP (BY22878)

The allele *SPC110*::*DsRed*-*KanMX* of strain TYY115-2D^46^ was amplified by PCR using primers SPC110DsRed-F (5′-GATGATGAACTAGATCGTGATTACTACAAT-3′) and SPC110DsRed-R (5′-ATATACCACATACATAGATATACCCTACGT-3′). The PCR fragment was introduced into YPH250.

To construct the strains carrying *TUB1*::*GFP*-*URA3*, pAFS125^47^ was digested with StuI, and the linearized fragment was integrated into the locus of *TUB1*.

To add a GFP tag at the C terminus of Nup116, YIp-*NUP116*-GFP^48^ or pRS304-*NUP116*-GFP^10^ was digested with EcoRI, and the linearized fragment was introduced into the locus of *NUP116*.

### Plasmids

Plasmids used in this study are summarized in Table 3. The plasmids pRS415-*GFP*-*ATG18* and pRS415-*GFP*-*ATG18*-*PHO8* (ALP) were constructed as follows: the ORF of *ATG18* was amplified with the following primers: ATG18-F-SpeI (5′-TTTACTAGTATGTCTGATTCATCACCTACTATCAA-3′) and ATG18-R-XbaI-XhoI (5′-TTTCTCGAGTCATCTAGAATCCATCAAGATGGAAT-3′). The PCR product was digested with SpeI and XhoI, and the resultant fragment was introduced into the XbaI and XhoI sites of pRS415-*MET25prom*-*GFP* (laboratory stock), which is the plasmid for the expression of GFP-N-terminal tagged proteins under *MET25* promoter. The resultant plasmid (pRS415-*GFP*-*ATG18*) was digested with XbaI (the stop codon of *ATG18* was deleted), and the ORF of *PHO8*, which was amplified by PCR using primers PHO8-F-XbaI (5′-AAATCTAGAATGATGACTCACACATTACCAAGCGA-3′) and PHO8-R-XbaI (5′-AAATCTAGATCAGTTGGTCAACTCATGGTAGTATT-3′), was ligated in-frame into the XbaI site.

**Table 3:** List of plasmids used in this study.

Plasmid	Description	Source/Reference
YCp50-*PKC1*^*R398P*^ pAFS125 YIp-*NUP116*-GFP pRS304-*NUP116*-GFP pKL2698 MET25p-GFP-*ATG18* MET25p-GFP-*ATG18-ALP* pRS416-*ATG18* pRS416-*ATG18*^*FTTG*^ pFL39-*PKC1*^*R398P*^	YCp50 (CEN type, *URA3* marker) harboring *PKC1*^*R398P*^ *TUB1-GFP* in an integrate-type, *URA3* marker plasmid *NUP116-GFP* in an integrate-type, *URA3* marker plasmid *NUP116-GFP* in an integrate-type, *TRP1* marker plasmid pRS316 (CEN type, *URA3* marker) harboring *SWE1* pRS415 (CEN type, *LEU2* marker) harboring GFP-*ATG18* pRS415 harboring GFP-*ATG18-ALP* pRS416 (CEN type, *URA3* marker) harboring *ATG18* pRS416 harboring *ATG18*^*FTTG*^ pFL39 (CEN type, *TRP1* marker) harboring *PKC1*^*R398P*^	^50^ ^47^ ^48^ ^10^ ^54^ This study This study This study This study This study

The plasmids pRS416-*ATG18* was constructed as follows: the genomic fragment containing *ATG18* was amplified with the following primers: ATG18-F-XhoI (5′-TTGCTCGAGACCATCTGACACATGTACACAGTAAC-3′) and ATG18-R-SacI (5′-TTAGAGCTCCCTGATTCATCTATTAGCCGTATAGA-3′). The PCR product was digested with XhoI and SacI, and the resultant fragment was introduced into the XhoI and SacI sites of pRS416^49^. pRS416-*ATG18*^*FTTG*^, the substitution of two Arg residues (Arg285 and Arg286) with Thr, was constructed using a KOD -Plus- Mutagenesis Kit (TOYOBO, Osaka, Japan) with pRS416-*ATG18* as a template.

To construct pFL39-*PKC1*^*R398P*^, YCp50-*PKC1*^*R398P*^^50^ was digested with SphI. The DNA fragment containing a *PKC1*^*R398P*^ was cloned into the SphI site of pFL39.

### Measurement of nuclear division

Cells were cultured in SD medium until the *A*_610_ reached 0.3–0.5, and 1 µg/ml Hoechst 33342 (Hoechst AG; Molecular Probes. Inc., Eugene, OR, USA) was added to stain the nucleus. To quantify the distribution of nuclei, cells with a large bud (bud diameter approximately two-thirds of that of the mother cell) containing the nucleus were counted after acquiring images with a fluorescence microscope, as reported previously^10^. Approximately 100–200 cells were counted for each experiment.

### Vacuolar staining

Yeast vacuoles were visualized in vivo by labelling with FM4-64 (Molecular Probes. Inc., Eugene, OR, USA) as reported previously^51^.

### Western blotting of Swe1

Preparation of total protein extracts using a Beads Smash 21 cell disrupter (Wakenyaku, Kyoto, Japan) were described previously^9^. The phosphorylation status of Tyr19 of Cdc28 and the amount of Cdc28 protein were detected using anti-phospho-Cdc2 antibody (#9,111, Cell Signalling Technology, MA, USA) and anti-Cdc2 p34 antibody (sc-53, Santa Cruz Biotechnology, CA, USA), respectively. Immunoreactive bands were visualized using a peroxidase stain kit (Nacalai tesque, Kyoto, Japan).

### Fluorescence microscopy

The fluorescence microscopes, BX51 and BX63 (Olympus equipped with the digital cameras DP70 (OLYMPUS, Tokyo, Japan) and ORCA-R2 (Hamamatsu Photonics, Shizuoka, Japan) were used.

### Analysis of phosphatidylinositols

Phosphatidylinositols (PtdIns3*P*_,_ PtdIns4*P*_,_ PtdIns(3,5)*P*_2_, and PtdIns(4,5)*P*_2_) were separated with HPLC and were measured as reported previously^11,52^.

### Statistical analysis

Data were presented as the means and standard deviation. The statistical significance of differences was evaluated using Student’s *t*-test. Differences with *p* values of < 0.05 were considered significant.

## Supplementary information

Supplementary file1.

## Abbreviations

MG: Methylglyoxal
PtdIns(3,5)*P*_2_: Phosphatidylinositol 3,5-bisphosphate
SD: Synthetic dextrose
SPB: Spindle pole body
