# Supplementary material for: Methylglyoxal inhibits nuclear division through alterations in vacuolar morphology and accumulation of Atg18 on the vacuolar membrane in Saccharomyces cerevisiae
Source: Sci Rep. 2020 Aug 17;10:13887. doi: 10.1038/s41598-020-70802-8 (PMC7431575; doi:10.1038/s41598-020-70802-8)

Methylglyoxal inhibits nuclear division through alterations in vacuolar morphology and accumulation of Atg18 on the vacuolar membrane in *Saccharomyces cerevisiae*

Wataru Nomura[1,2]*, Miho Aoki[1], and Yoshiharu Inoue[1]*

[1] Laboratory of Molecular Microbiology, Division of Applied Life Sciences, Graduate School of Agriculture, Kyoto University, Uji, Kyoto 611-0011, Japan

[2] Present address: Laboratory of Molecular Function of Food, Division of Food Science and Biotechnology, Graduate School of Agriculture, Kyoto University, Uji, Kyoto 611-0011, Japan

FIGURE 4(C) and (B)

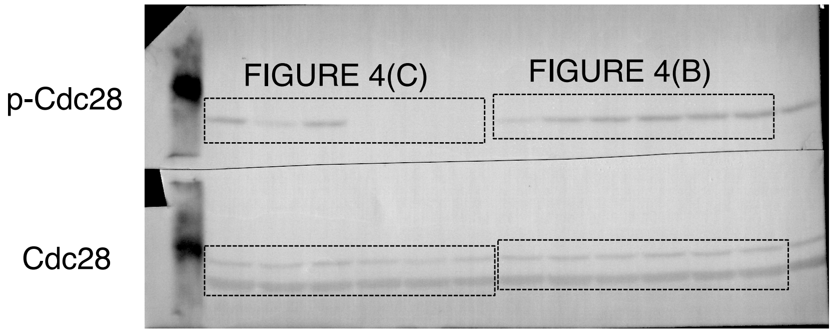

FIGURE 4(F)

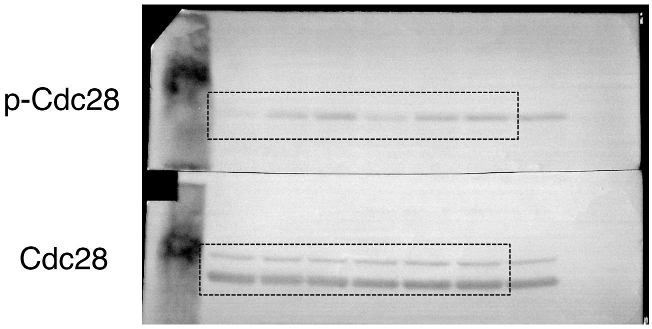

Supplement: Supplementary file 1 — Supplementary file1. [file 41598_2020_70802_MOESM1_ESM.pdf]
